# Resveratrol ameliorates maternal immune activation-associated cognitive impairment in adult male offspring by relieving inflammation and improving synaptic dysfunction

**DOI:** 10.3389/fnbeh.2023.1271653

**Published:** 2023-11-22

**Authors:** Yue-Ming Zhang, Ru-Meng Wei, Meng-Ying Zhang, Kai-Xuan Zhang, Jing-Ya Zhang, Shi-Kun Fang, Yi-Jun Ge, Xiao-Yi Kong, Gui-Hai Chen, Xue-Yan Li

**Affiliations:** ^1^Department of Neurology (Sleep Disorders), The Affiliated Chaohu Hospital of Anhui Medical University, Hefei, Anhui, China; ^2^Department of Anesthesiology, The Affiliated Chaohu Hospital of Anhui Medical University, Hefei, Anhui, China

**Keywords:** maternal immune activation, resveratrol, learning and memory, SIRT1, inflammation, synaptic plasticity

## Abstract

Maternal exposure to inflammation may represent a major risk factor for neuropsychiatric disorders with associated cognitive dysfunction in offspring in later life. Growing evidence has suggested that resveratrol exerts a beneficial effect on cognitive impairment via its anti-inflammatory and antioxidant properties and by ameliorating synaptic dysfunction. However, how resveratrol affects maternal immune activation-induced cognitive dysfunction and the underlying mechanisms are unclear. In the present study, pregnant dams were given an intraperitoneal injection of lipopolysaccharide (LPS; 50 μg/kg) on gestational day 15. Subsequently, the offspring mice were treated or not with resveratrol (40 mg/kg) from postnatal day (PND) 60 to PND 88. Male offspring were selected for the evaluation of cognitive function using the Morris water maze test. The hippocampal levels of pro-inflammatory cytokines were examined by ELISA. The mRNA and protein levels of sirtuin-1 (SIRT1), brain-derived neurotrophic factor (BDNF), postsynaptic density protein 95 (PSD-95), and synaptophysin (SYP) were determined by RT-qPCR and western blot, respectively. The results showed that male offspring mice exposed to LPS *in utero* exhibited learning and memory impairment. Additionally, the levels of interleukin (IL)-1β, IL-6, and tumor necrosis factor-alpha (TNF-α) were increased while those of SIRT1, BDNF, PSD-95, and SYP were decreased in male offspring of LPS-treated mothers. Treatment with resveratrol reversed cognitive impairment and attenuated the increase in the levels of pro-inflammatory cytokines induced by maternal immune activation in the offspring mice. Furthermore, resveratrol reversed the deleterious effects of maternal immune activation on SIRT1, BDNF, PSD-95, and SYP levels in the hippocampus. Collectively, our results suggested that resveratrol can effectively improve learning and memory impairment induced by maternal immune activation via the modulation of inflammation and synaptic dysfunction.

## 1 Introduction

Over the past few decades, a growing body of studies has explored the role of the intrauterine environment in structuring fetal development and how it influences the mental and physical health of offspring later in life (O’Donnell and Meaney, 2017). The maternal immune activation hypothesis proposes that pregnant women are susceptible to inflammation owing to physical and psychological factors such as chronic immune disorders, infection, or stress and that maternal inflammation may affect the neurodevelopment of the fetus (Segovia et al., 2017; Osborne et al., 2019; Han et al., 2021). Epidemiological reports have shown that maternal inflammation during pregnancy is a major risk factor for neurological and psychiatric disorders, including cognitive dysfunction, schizophrenia, depression, and autism, later in the life of the offspring, resulting in behavioral abnormalities (Knuesel et al., 2014). Lipopolysaccharide (LPS), found on the outer membrane of Gram-negative bacteria, is commonly used to create models of maternal gestational infection in basic research. Its use has revealed that maternal inflammation leads to cognitive impairment in offspring mice (Kentner et al., 2019; Zhuang et al., 2020; Fernandez de Cossio et al., 2021) and that the underlying mechanisms may be related to pro-inflammatory/anti-inflammatory system imbalance, epigenetic inheritance, and dysregulation of the hypothalamic–pituitary–adrenal axis (Oldenburg et al., 2020; Rahimi et al., 2020). However, the mechanisms involved in how exposure to inflammation during pregnancy subsequently leads to cognitive impairment in offspring remain largely unknown.

Inflammation has been reported to play an essential role in mediating cognitive deficits in offspring exposed to inflammation *in utero* (Kuypers et al., 2013). In rodents, exposure to LPS during pregnancy stimulates the production of pro-inflammatory cytokines by the mother; these cytokines can cross the placental barrier, leading to an increase in their concentrations in the fetal brain and amniotic fluid, and resulting in impaired fetal neurodevelopment and cognitive behavioral abnormalities in offspring later in life (Kwon et al., 2022). One study demonstrated that the mechanism underlying prenatal inflammation-mediated cognitive dysfunction in elderly offspring mice involves an increase in the levels of the pro-inflammatory cytokines interleukin (IL)-1β, IL-6, and tumor necrosis factor-alpha (TNF-α) (Zhang et al., 2022).

Early-life stress can result in synaptic dysfunction, which is a major cause of subsequent cognitive impairment (Bock et al., 2015). Postsynaptic density protein 95 (PSD-95) is a represented member of the PSD protein family that contributes to the maintenance of synaptic connectivity and also plays a role in synaptic plasticity (Murack et al., 2023). Synaptophysin (SYP) is a key constituent of the presynaptic vesicle membrane and is involved in the regulation of dendritic and axonal growth and differentiation, and neurotransmitter secretion (Zhang et al., 2014). Both PSD-95 and SYP are specific markers of synapses and are important for proper cognitive function (Liu et al., 2018).

It has been shown that early life stresses, such as maternal lead or alcohol exposure, can lead to decreased levels of synaptic proteins, including PSD-95 and SYP, resulting in impaired synaptic plasticity and changes in the synaptic ultrastructure in the hippocampus, finally leading to cognitive dysfunction in offspring rodents (Elibol-Can et al., 2014; Gąssowska et al., 2016). Brain-derived neurotrophic factor (BDNF), mainly distributed in the central nervous system, functions in the promotion of neuronal growth, neuronal synapse formation and stabilization, and long-term potentiation and is essential for regulating memory-related neuroplasticity (Palasz et al., 2020). Both clinical and basic studies have shown that BDNF levels are significantly reduced in disorders associated with cognitive decline (Liu et al., 2020; Gaitan et al., 2021). Combined, these observations suggest that neuroinflammation and synaptic plasticity-associated proteins, such as PSD-95, SYP, and BDNF, may be involved in the cognitive impairment of offspring induced by maternal inflammation during pregnancy.

Over recent years, resveratrol, a natural sirtuin-1 (SIRT1) agonist mainly derived from peanuts, grapes, and berries, has received attention for its neuroprotective effects (Yang et al., 2021). Resveratrol exerts positive effects on inflammation, synaptic dysfunction, and oxidative stress through the activation of the SIRT1 gene, thus contributing to improving cognitive function (Gomes et al., 2018). Clinical studies have shown that supplementation with this polyphenolic substance can enhance immunity and reduce neuroinflammation, thereby improving cognition and lowering the risk of dementia (Moussa et al., 2017; Pyo et al., 2020; Buglio et al., 2022). Similarly, preclinical studies have shown that resveratrol can ameliorate isoflurane-induced cognitive dysfunction in aged mice by relieving neuroinflammation via the activation of SIRT1 (Li et al., 2014). Meanwhile, resveratrol was reported to attenuate Pb- and Aβ1–42-induced learning memory impairments by activating SIRT1, thus exerting a protective effect on hippocampal synaptic function (Wang et al., 2017, 2021). These observations suggest that resveratrol may have the potential to ameliorate maternal inflammation-induced cognitive deficits in adult offspring by improving neuroinflammation and synaptic dysfunction through the activation of SIRT1.

In the present study, using male C57BL/6 J mice, we explored whether resveratrol exerts ameliorative effects on cognitive impairment in adult offspring induced by prenatal exposure to inflammation and, if so, whether these effects were mediated through the activation of SIRT1.

## 2 Materials and methods

### 2.1 Animals

C57BL/6 J mice were purchased from Beijing Vital River Laboratory Animal Device Co., Ltd. and were housed under controlled conditions (relative humidity, 50 ± 5%; temperature, 22–25°C; 12 h light/12 h dark schedule [lights on at 08:00 h]) and had *ad libitum* access to food and water. Mating was performed at a 1:2 male-to-female ratio. The time of pregnancy was confirmed by the vaginal plug through visual inspection and was identified as day 0. All experimental procedures were approved by the Laboratory Animal Committee of Anhui Medical University.

### 2.2 Treatments

**Figure 1:**
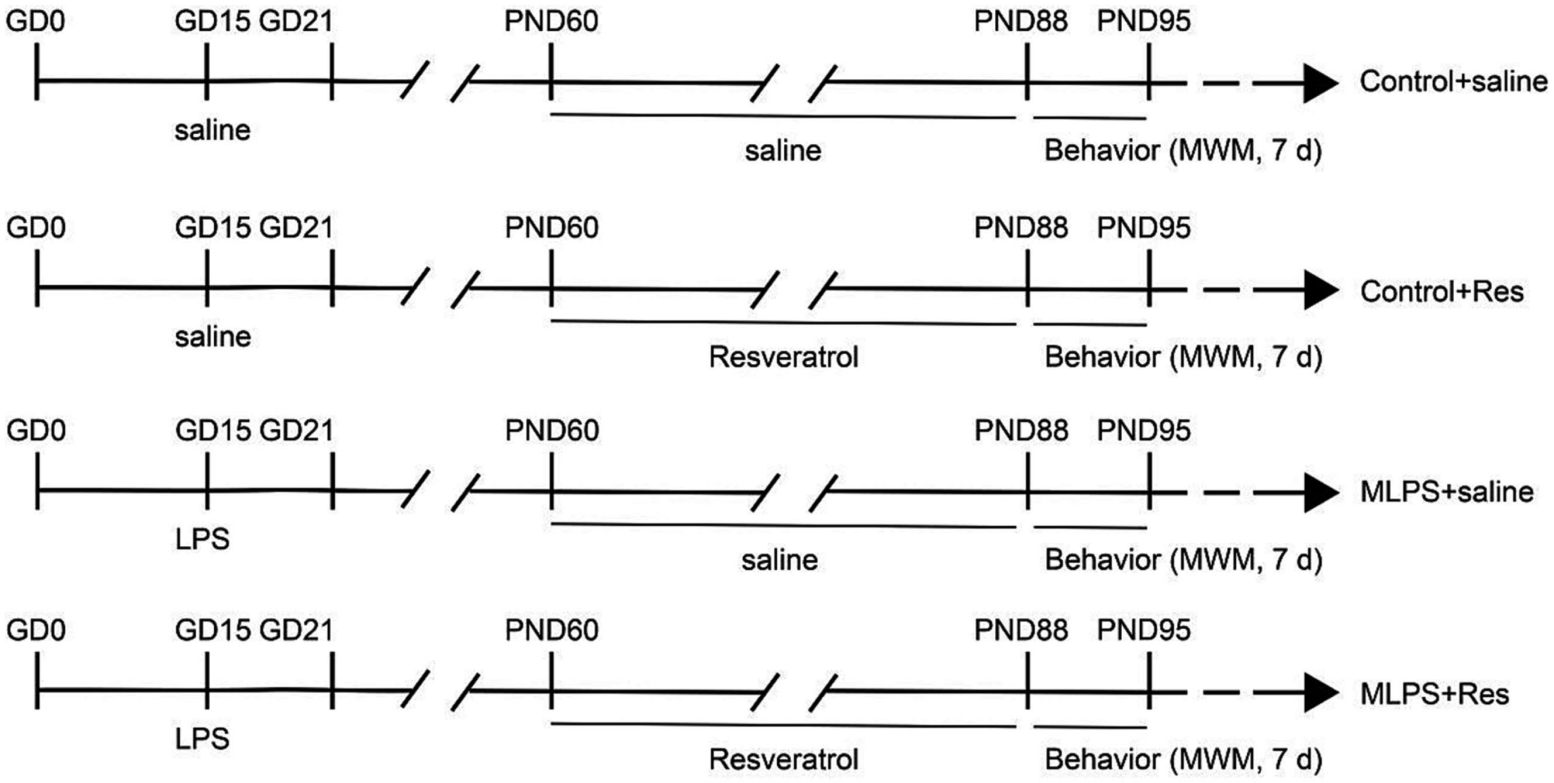
Experimental protocol. GD, gestational day; LPS, lipopolysaccharide; PND, postnatal day; MWM, Morris water maze; d, days.

A diagram of the treatment schedule is shown in Figure 1. On day 15 of gestation, pregnant mice received either an intraperitoneal injection of LPS (Abcam LPS, Shanghai Universal Biotech Co., Ltd., China) with a dose of 50 μg/kg or saline. The dose of LPS was chosen based on previous studies by our group (Zhang et al., 2020, 2022). The day of delivery was designated as postnatal day 0 (PND 0). On PND 21, the male offspring were selected by randomly choosing one mouse from each litter and divided into the following groups (*n* = 8 per group; the offspring mice were randomly selected from each litter): a Control+saline group; a Control+Resveratrol group; an MLPS group, comprising offspring of LPS-treated mothers; and an MLPS + Resveratrol group. At 2 months of age, offspring mice in the Control+saline and MLPS + Resveratrol groups received a daily intraperitoneal injection of resveratrol [40 mg/(kg/day^−1^)] for 4 weeks. We chose 40 mg/kg/day of resveratrol based on previous studies showing that this dose is the appropriate dose to ameliorate stress-induced hippocampal inflammation and cognitive deficits and to ensure maximal efficacy while decreasing the occurrence of side effects (Shen et al., 2018; Wang F. et al., 2020). Resveratrol was first dissolved in dimethyl sulfoxide (DMSO, Absin, abs9189) and then in a saline solution containing 5% Tween 80 (Abbexa, abx082610). The vehicle consisted of 5% DMSO and 5% Tween 80 in saline. All behavioral experiments were performed between 13:00 and 18:00 h (see Figure 1). All experimental procedures were approved by the Laboratory Animal Committee of Anhui Medical University.

### 2.3 Morris water maze test

The MWM test was performed as previously reported (Zhang et al., 2022). The offspring were trained four times a day for 7 consecutive days, with an interval of 15 min between training sessions. Each trial lasted 60 s and the mice were placed in a different quadrant of the tank for each trial. The latency, distance, and swimming velocity were recorded using a video camera and analyzed using ANY-Maze software (Stoeling, United States). Probe trials, in which the hidden platform was removed, were performed shortly after the end of the training phase on day 7 of the experiment. The mice were placed in the tank and were allowed to freely explore for 60 s. The percentage of time and the distance in the target quadrant were also determined with ANY-Maze software (Stoeling).

### 2.4 Tissue preparation

After the MWM tests, the offspring mice were deeply anesthetized with 2% sodium pentobarbital. Hippocampal tissue was dissected from the animals and placed at −80°C for enzyme-linked immunosorbent assay (ELISA), western blot, and RT-PCR analysis.

### 2.5 Elisa

The levels of the pro-inflammatory cytokines IL-1β, TNF-α, and IL-6 in hippocampal tissue were measured using the respective ELISA kits (JYM0531Mo, JYM0218Mo, and JYM0012Mo; Wuhan Colorful Biotechnology Co., Wuhan, China) according to the manufacturer’s instructions.

### 2.6 Real-time quantitative fluorescence PCR

Total RNA was extracted from the hippocampal tissue using TRIzol reagent following the manufacturer’s instructions. The extracted RNA was reverse transcribed to cDNA using the PrimeScript RT Reagent Kit with gDNA Eraser (Takara, RR047A). The cDNA was used as a template for fluorescence-based qualification. The qPCR cycling conditions were 95°C for 1 min, followed by 40 cycles of 95°C for 20 s and 60°C for 1 min. Relative mRNA levels were quantified using the 2^−∆∆Ct^ method. The primer sequences are shown in Table 1.

**Table 1:** Primer sequences used for RT-PCR.

Gene	Amplicon size (bp)	Forward primer (5′ → 3′)	Reverse primer (5′ → 3′)
β-actin	120	AGTGTGACGTTGACATCCGT	TGCTAGGAGCCAGAGCAGTA
BDNF	94	TTACTCTCCTGGGTTCCTGA	ACGTCCACTTCTGTTTCCTT
PSD-95	110	GCTCCCTGGAGAATGTGCTA	TGAGAAGCACTCCGTGAACT
SYN	124	GCCTACCTTCTCCACCCTTT	GCACTACCAACGTCACAGAC
SIRT1	116	TAATGTGAGGAGTCAGCACC	GCCTGTTTGGACATTACCAC

### 2.7 Western blotting

Hippocampal tissue was homogenized in RIPA lysis buffer and centrifuged at 12,000 rpm for 15 min. The resulting supernatant was mixed with 5× SDS–PAGE protein loading buffer (1:4) and the proteins were denatured in a boiling water bath for 15 min. Subsequently, the proteins were resolved using SDS–PAGE at a constant voltage (80 V) for approximately 1 h, transferred onto PVDF membranes, rinsed, blocked, and incubated with rabbit antibodies against PSD-95 (1:2,000; ab238135, Abcam), SYP (1:1,000; bs-8845R, Bioss), and BDNF (1:1,000; ab108319, Abcam) and a mouse antibody targeting SIRT1 (1,4,000; ab110304, Abcam) overnight at 4°C. The membranes were incubated with a horseradish peroxidase-labeled secondary antibody (120,000; ZB-2301, Zsbio) after washing. The protein bands were quantified by densitometry using ImageJ software (Media Cybernetics, United States).

### 2.8 Statistical analyses

The results of this research were analyzed in GraphPad Prism 8.0. Differences in markers of inflammation and synaptic plasticity were analyzed using a two-way analysis of variance (ANOVA) followed by Tukey’s *post-hoc* test. The significance of the MWM test results was assessed using repeated measures ANOVA followed by Tukey’s *post-hoc* test. Pearson’s correlation coefficient was used to assess correlations. *p* < 0.05 was considered significant.

## 3 Results

### 3.1 Resveratrol improved learning and memory impairment in offspring mice of LPS-treated mothers

**Figure 2:**
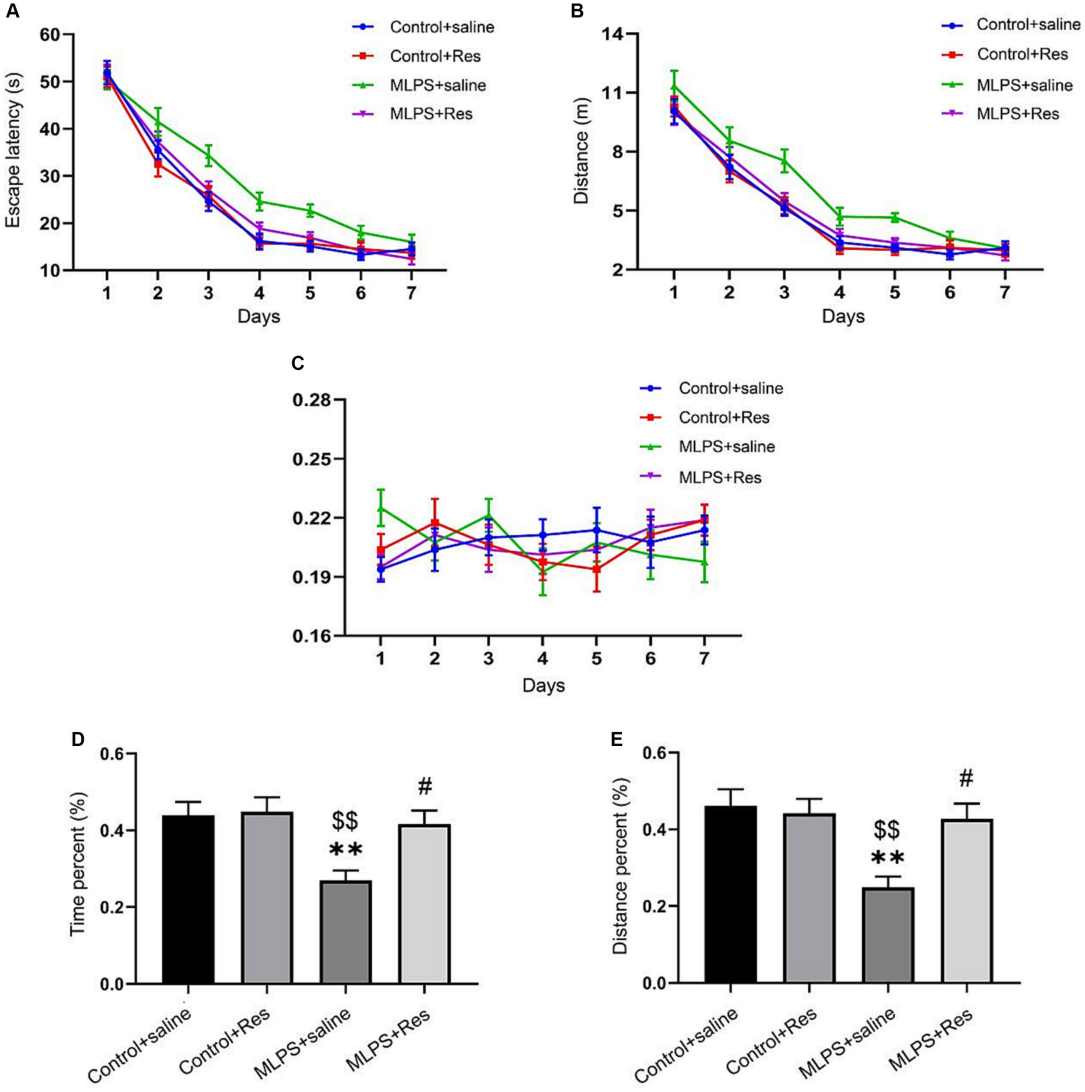
The effects of resveratrol (Res) on maternal lipopolysaccharide (LPS) exposure-induced spatial learning and memory deficits of offspring mice in the Morris water maze test. **(A)** Escape latency for each group of mice in the seven training days. **(B)** Distance swam for each group of mice in the seven training days. **(C)** Swimming velocity for each group of mice in the seven training days. **(D)** The percentage of time in the target quadrant for each group of mice during the probe test. **(E)** The percentage of distance in the target quadrant for each group of mice during the probe test. ^**^p < 0.01 compared to the Control + saline group; ^$$^*p* < 0.01 compared to the Control + Res group; ^#^*p* < 0.05 compared to the maternal LPS exposure (MLPS) + saline group.

The effects of resveratrol on LPS-induced learning and memory impairment were assessed on PND 90 using the MWM test. In the acquisition phase, repeated measures ANOVA showed significant effects of treatment and time for both the escape latency and the distance swam (escape latency: *F*_(3,28)_ = 20.08, *p* < 0.01; distance: *F*_(3,28)_ = 13.84, *p* < 0.01; Figures 2A,B). However, no effect was observed for the interaction between time and treatment (escape latency: *F*_(18,168)_ = 1.10, *p* > 0.05; distance: *F*_(18,168)_ = 0.76, *p* > 0.05; Figures 2A,B). *Post-hoc* comparisons indicated that, compared with the Control group, the escape latency and the distance swam were markedly increased in the MLPS group; however, this spatial memory deficit was significantly reversed by resveratrol treatment. No difference in swimming velocity was found among the four groups (Figure 2C).

In the retention phase, the time and distance percent in the target quadrant differed significantly among the four groups (time percent: *F*_(3,28)_ = 6.36, *p* < 0.01; distance percent: *F*_(3,28)_ = 6.94, *p* < 0.01; Figures 2D,E). Further analysis revealed that the percentage of the time and distance in the target quadrant in the MLPS group was decreased compared with that seen in the Control or Resveratrol groups. However, these negative effects caused by LPS exposure were improved by resveratrol treatment. Together, these results indicated that resveratrol treatment can ameliorate LPS-induced learning and memory impairment.

### 3.2 Resveratrol inhibited the LPS-induced pro-inflammatory response in the hippocampus of offspring mice

**Figure 3:**
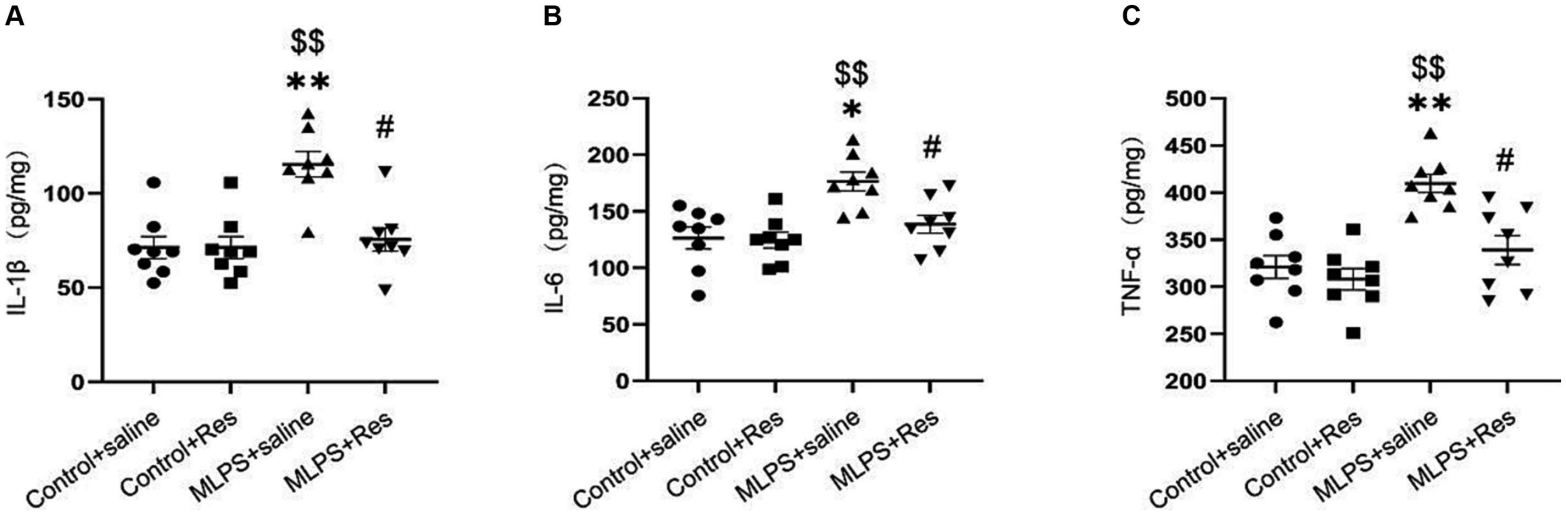
The effect of resveratrol (Res) on the IL-1β, IL-6, and TNF-α in the hippocampus of offspring mice exposed to lipopolysaccharide (LPS) *in utero*. **(A)** The level of IL-1β. **(B)** The level of IL-6. **(C)** The level of TNF-α. ^*^p < 0.05, ^**^p < 0.01 compared to the Control + saline group; ^$$^*p* < 0.01 compared to the Control + Res group; ^#^*p* < 0.05 compared to the maternal LPS exposure (MLPS) + saline group.

To evaluate the potential of resveratrol in ameliorating the maternal LPS exposure-induced inflammatory response in offspring mice, we measured the levels of various pro-inflammatory cytokines (IL-1β, IL-6, and TNF-α) in the hippocampus. The concentrations of the three cytokines were different among the four groups (IL-1β: *F*_(3,28)_ = 12.20, *p* < 0.01; IL-6: *F*_(3,28)_ = 8.46, *p* < 0.01; TNF-α: *F*_(3,28)_ = 13.49, *p* < 0.01; Figures 3A–C). The *post-hoc* analysis showed that maternal LPS exposure increased the hippocampal levels of IL-1β, IL-6, and TNF-α in offspring mice (*Ps* < 0.05), whereas resveratrol treatment suppressed this effect (*Ps* < 0.05).

In summary, these findings confirmed that resveratrol can greatly inhibit the maternal LPS exposure-induced pro-inflammatory response in the hippocampus of offspring mice.

### 3.3 Resveratrol elevated the expression of BDNF, SIRT1, PSD-95, and SYP in the hippocampus

**Figure 4:**
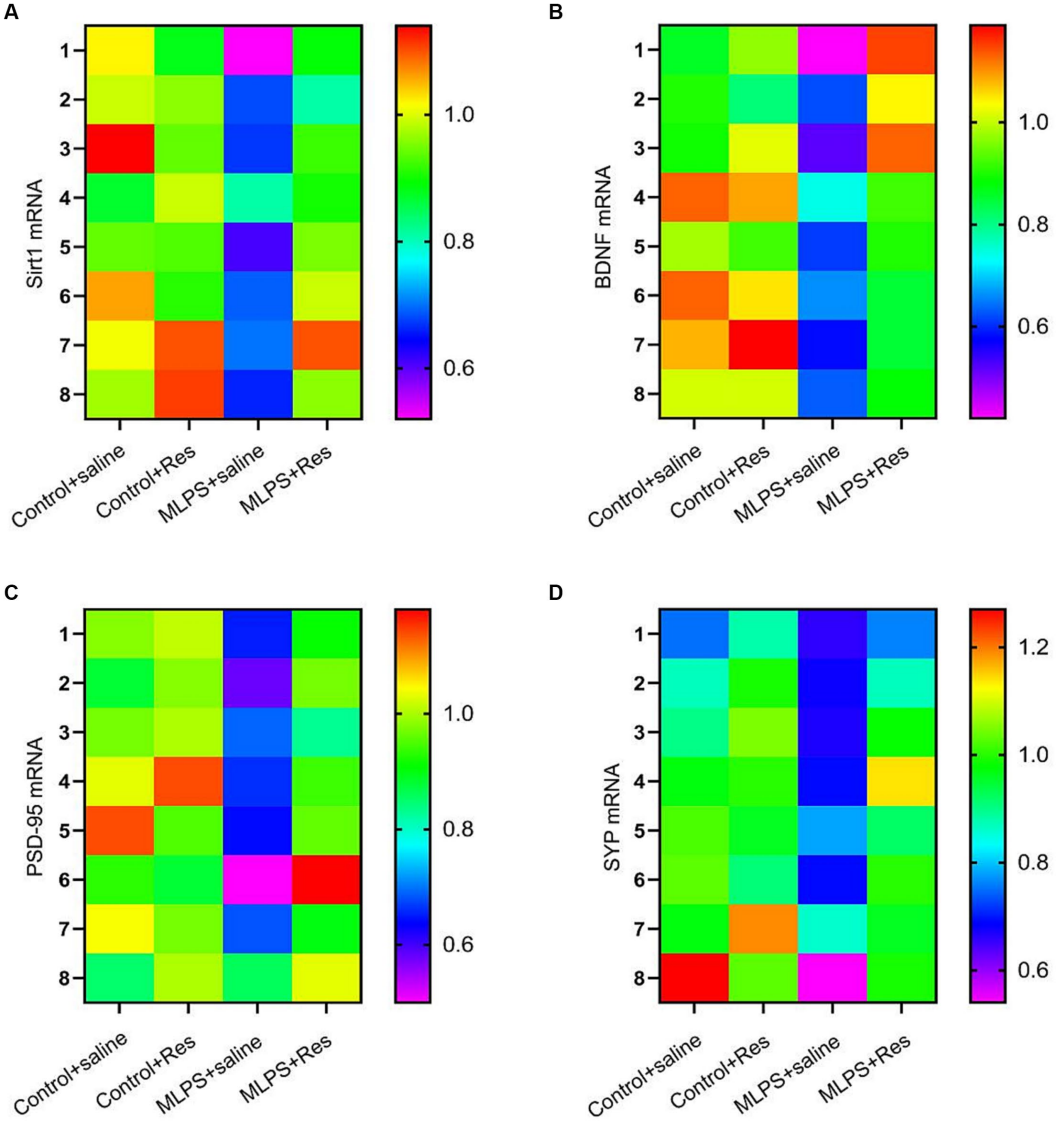
The effects of resveratrol (Res) on the mRNA levels of SIRT1, BDNF, PSD-95, and SYP in the hippocampus of offspring mice of mothers exposed to lipopolysaccharide (LPS) during pregnancy. Heat map of the mRNA levels of SIRT1, BDNF, PSD-95, and SYP **(A–D)** in the hippocampus.

**Figure 5:**
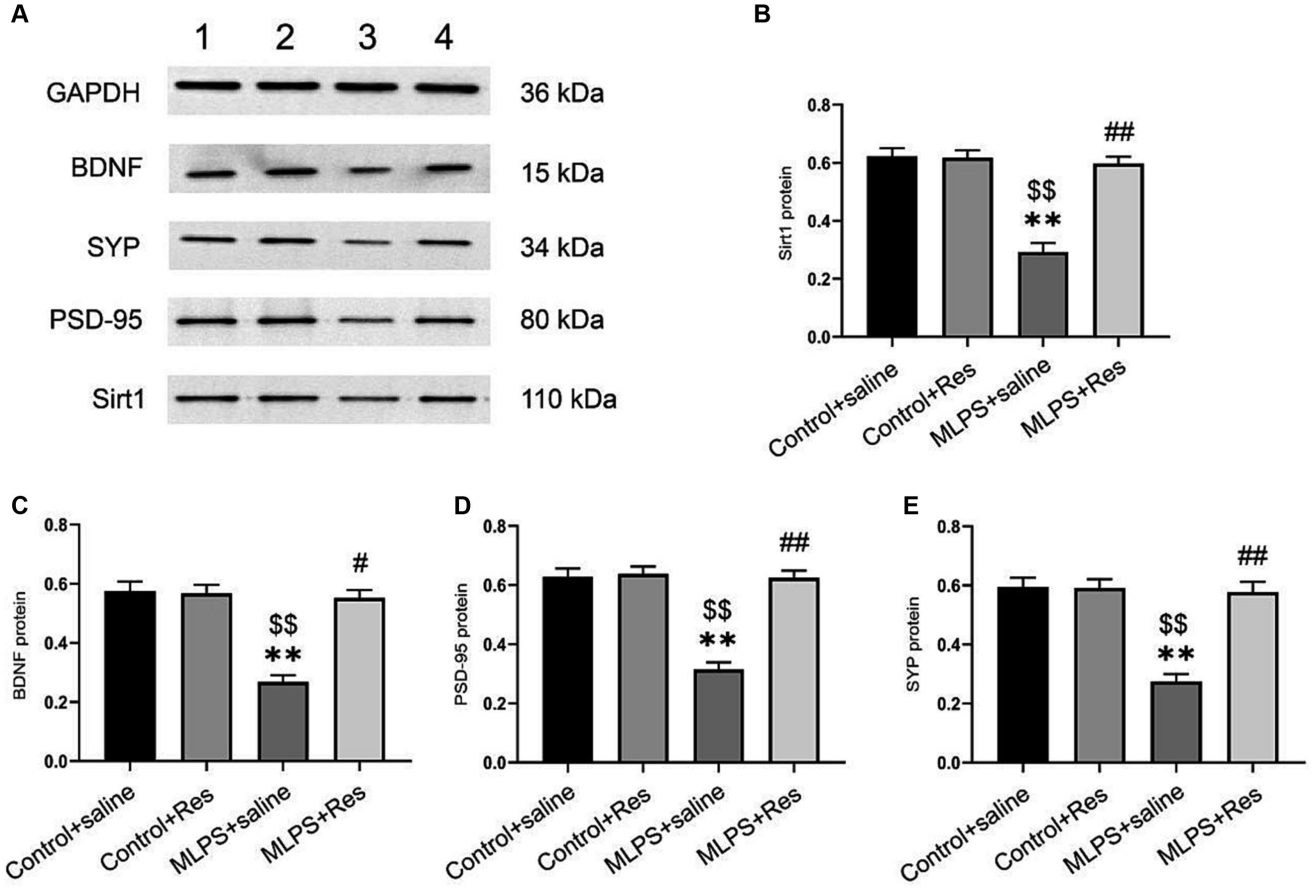
The effects of resveratrol (Res) on the protein expression levels of SIRT1, BDNF, PSD-95, and SYP in the hippocampus of offspring mice exposed to lipopolysaccharide (LPS) *in utero*. **(A)** Representative western blots of SIRT1, BDNF, PSD-95, and SYP in the hippocampus: band 1, Control+saline group; band 2, Control+Res group; band 3, MLPS+saline group; band 4, MLPS+Res group. **(B)** Quantitative analysis of SIRT1 levels. **(C)** Quantitative analysis of BDNF levels. **(D)** Quantitative analysis of PSD-95 levels. **(E)** Quantitative analysis of SYP levels. ^**^p < 0.01 compared to the Control + saline group; ^$$^*p* < 0.01 compared to the Control + Res group; ^#^*p* < 0.05, ^##^*p* < 0.01 compared to the maternal LPS exposure (MLPS) + saline group.

We investigated the effect of resveratrol and maternal LPS treatment on BDNF, SIRT1, PSD-95, and SYP levels in the hippocampus of offspring mice. One-way ANOVA identified a significant effect of treatment on the expression levels of BDNF, SIRT1, PSD-95, and SYP in the hippocampus (mRNA: BDNF: *F*_(3,28)_ = 25.35, *p* < 0.01; SIRT1: *F*_(3,28)_ = 27.69, *p* < 0.01; PSD-95: *F*_(3,28)_ = 23.21, *p* < 0.01; SYP: *F*_(3,28)_ = 12.10, *p* < 0.01; Figures 4A–D; protein: BDNF: *F*_(3,20)_ = 30.69, *p* < 0.01; SIRT1: *F*_(3,20)_ = 35.95, *p* < 0.01; PSD-95: *F*_(3,20)_ = 38.91, *p* < 0.01; SYP: *F*_(3,20)_ = 26.82, *p* < 0.01; Figures 5A–E). *Post-hoc* analysis demonstrated that the learning and memory impairment induced by *in-utero* exposure to LPS (MLPS group) resulted in decreased mRNA and protein levels of BDNF, SIRT1, PSD-95, and SYP (*Ps* < 0.05); however, this effect was not detected with resveratrol treatment (*Ps* < 0.05).

### 3.4 Correlations between cognitive impairment-like behaviors and hippocampal expression levels of pro-inflammatory cytokines

In the acquisition phase of the MWM test, the hippocampal IL-1β, IL-6, and TNF-α were positively correlated with the distance swam and the latency in the four groups (*Ps* < 0.05; Table 2). In the retention phase, the time and distance percent in the target quadrant were negatively correlated with the hippocampal levels of the three pro-inflammatory factors in the four groups (*Ps* < 0.05; Table 2).

**Table 2:** Correlations between the performance in the Morris water maze test and the hippocampal levels of IL-1β, IL-6, and TNF-α [r (p)].

Tasks	Indexes	Groups	Pro-inflammatory cytokines		
			IL-1β	IL-6	TNF-α
Morris water maze test	Escape latency	Control+saline	0.438 (0.278)	0.521 (0.186)	0.645 (0.084)
		Control+Res	0.860 (0.006)**	0.871 (0.005)**	0.921 (0.001)**
		MLPS+saline	0.913 (0.002)**	0.825 (0.012)*	0.862 (0.006)**
		MLPS+Res	0.825 (0.012)*	0.889 (0.003)**	0.773 (0.024)*
	Distance swam	Control+saline	0.530 (0.177)	0.560 (0.149)	0.594 (0.121)
		Control+Res	0.724 (0.042)*	0.717 (0.045)*	0.814 (0.014)*
		MLPS+saline	0.876 (0.004)**	0.798 (0.018)*	0.785 (0.021)*
		MLPS+Res	0.727 (0.041)*	0.903 (0.002)**	0.852 (0.007)**
	Percentage of time swam	Control+saline	−0.555 (0.153)	−0.546 (0.161)	−0.658 (0.076)
		Control+Res	−0.894 (0.003)**	−0.882 (0.004)**	−0.932 (0.001)**
		MLPS+saline	−0.983 (0.000)**	−0.813 (0.014)*	−0.876 (0.004)**
		MLPS+Res	−0.826 (0.011)*	−0.928 (0.001)**	−0.860 (0.006)**
	Percentage of distance swam	Control+saline	−0.208 (0.621)	−0.088 (0.837)	−0.104 (0.807)
		Control+Res	−0.807 (0.016)*	−0.911 (0.002)**	−0.811 (0.015)*
		MLPS+saline	−0.957 (0.000)**	−0.785 (0.021)*	−0.829 (0.011)*
		MLPS+Res	−0.805 (0.016)*	−0.882 (0.004)**	−0.911 (0.002)**

Res, resveratrol; MLPS, maternal lipopolysaccharide (LPS) exposure. *p < 0.05; **p < 0.01.

### 3.5 Correlations between cognitive impairment-like behaviors and expression levels of BDNF, SIRT1, PSD-95, and SYP

#### 3.5.1 Correlations with the mRNA levels of BDNF, SIRT1, PSD-95, and SYP

The correlation analysis revealed that the escape latency and the distance swam in the acquisition phase of the MWM assay were negatively correlated with the hippocampal mRNA levels of BDNF, SIRT1, PSD-95, and SYP in the four groups (*Ps* < 0.05; Table 3). Additionally, the time and distance percent in the target quadrant in the retention phase of the test exhibited negative correlations with the levels of BDNF, SIRT1, PSD-95, and SYP mRNA in the four groups (*Ps* < 0.05; Table 3).

**Table 3:** Correlations between the performance in the Morris water maze test and the hippocampal mRNA levels of SIRT1, BDNF, PSD-95, and SYP [r (p)].

Tasks	Indexes	Groups	mRNA levels			
			SIRT1	BDNF	PSD-95	SYP
Morris water maze test	Escape latency	Control+saline	−0.454 (0.259)	−0.515 (0.191)	−0.342 (0.407)	−0.592 (0.122)
		Control+Res	−0.884 (0.004)**	−0.918 (0.001)**	−0.918 (0.001)**	−0.933 (0.001)**
		MLPS+saline	−0.892 (0.003)**	−0.855 (0.007)**	−0.881 (0.004)**	−0.900 (0.002)**
		MLPS+Res	−0.939 (0.001)**	−0.908 (0.002)**	−0.938 (0.001)**	−0.891 (0.003)**
	Distance swam	Control+saline	−0.589 (0.125)	−0.676 (0.066)	−0.511 (0.195)	−0.754 (0.031)*
		Control+Res	−0.862 (0.006)**	−0.866 (0.005)**	−0.749 (0.033)*	−0.808 (0.015)*
		MLPS+saline	−0.861 (0.006)**	−0.854 (0.007)**	−0.851 (0.007)**	−0.916 (0.001)**
		MLPS+Res	−0.862 (0.006)**	−0.823 (0.012)*	−0.802 (0.017)*	−0.893 (0.003)**
	Percentage of time swam	Control+saline	0.550 (0.158)	0.687 (0.060)	0.435 (0.282)	0.769 (0.026)*
		Control+Res	0.892 (0.003)**	0.929 (0.001)**	0.951 (0.000)**	0.947 (0.000)**
		MLPS+saline	0.972 (0.000)**	0.904 (0.002)**	0.989 (0.000)**	0.966 (0.000)**
		MLPS+Res	0.871 (0.005)**	0.966 (0.000)**	0.939 (0.001)**	0.852 (0.007)**
	Percentage of distance swam	Control+saline	0.102 (0.809)	0.666 (0.071)	0.091 (0.831)	0.441 (0.274)
		Control+Res	0.819 (0.013)*	0.845 (0.008)**	0.846 (0.008)**	0.853 (0.007)**
		MLPS+saline	0.877 (0.004)**	0.730 (0.040)*	0.932 (0.001)**	0.874 (0.005)**
		MLPS+Res	0.804 (0.016)*	0.865 (0.006)**	0.935 (0.001)**	0.830 (0.011)*

Res, resveratrol; MLPS, maternal lipopolysaccharide (LPS) exposure. *p < 0.05; **p < 0.01.

#### 3.5.2 Correlations with BDNF, SIRT1, PSD-95, and SYP protein levels

In the acquisition phase of the MWM test, the protein levels of BDNF, SIRT1, PSD-95, and SYP were negatively correlated with the latency and distance in the four groups (*Ps* < 0.05; Table 4). In the retention phase, the percent distance swam and the percent time spent in the target quadrant were positively correlated with the hippocampal levels of the four proteins in the four groups (*Ps* < 0.05; Table 4).

**Table 4:** Correlations between the performance in the Morris water maze test and the hippocampal levels of synaptic proteins [r (p)].

Tasks	Indexes	Groups	Levels of synaptic proteins			
			SIRT1	BDNF	PSD-95	SYP
Morris water maze test	Escape latency	Control+saline	−0.707 (0.116)	−0.644 (0.167)	−0.654 (0.159)	−0.730 (0.099)
		Control+Res	−0.842 (0.035)*	−0.899 (0.015)*	−0.826 (0.043)*	−0.944 (0.005)**
		MLPS+saline	−0.887 (0.019)*	−0.945 (0.004)**	−0.960 (0.002)**	−0.920 (0.009)**
		MLPS+Res	−0.918 (0.010)**	−0.880 (0.021)*	−0.876 (0.022)*	−0.903 (0.014)*
	Distance swam	Control+saline	−0.404 (0.427)	−0.617 (0.191)	−0.430 (0.395)	−0.566 (0.241)
		Control+Res	−0.928 (0.008)**	−0.918 (0.010)**	−0.888 (0.018)*	−0.962 (0.002)**
		MLPS+saline	−0.914 (0.011)*	−0.930 (0.007)**	−0.925 (0.008)**	−0.911 (0.011)*
		MLPS+Res	−0.855 (0.030)*	−0.867 (0.026)*	−0.910 (0.012)*	−0.834 (0.039)*
	Percentage of time swam	Control+saline	0.644 (0.167)	0.810 (0.051)	0.667 (0.148)	0.782 (0.066)
		Control+Res	0.852 (0.031)*	0.913 (0.011)*	0.832 (0.040)*	0.935 (0.006)**
		MLPS+saline	0.922 (0.009)**	0.985 (0.000)**	0.971 (0.001)**	0.956 (0.003)**
		MLPS+Res	0.858 (0.029)*	0.851 (0.032)*	0.880 (0.021)*	0.947 (0.004)**
	Percentage of distance swam	Control+saline	0.336 (0.515)	0.701 (0.121)	0.392 (0.442)	0.575 (0.232)
		Control+Res	0.892 (0.017)*	0.946 (0.004)**	0.893 (0.017)*	0.889 (0.018)*
		MLPS+saline	0.899 (0.015)*	0.950 (0.004)**	0.816 (0.048)*	0.938 (0.006)**
		MLPS+Res	0.988 (0.000)**	0.943 (0.005)**	0.897 (0.015)*	0.973 (0.001)**

Res, resveratrol; MLPS, maternal lipopolysaccharide (LPS) exposure. *p < 0.05; **p < 0.01.

## 4 Discussion

In this work, we found that maternal exposure to LPS on GD15 resulted in significant learning and memory deficits in offspring mice. These effects were accompanied by neuroinflammation and synaptic dysfunction, which may be associated with SIRT1/BDNF pathway dysregulation; however, resveratrol treatment ameliorated these negative effects of LPS, likely by upregulating SIRT1 expression.

### 4.1 Resveratrol improved learning and memory impairment induced by maternal immune activation

The perinatal period is a critical window in the development of mammals, including humans and mice, but it is also when early development is most susceptible to interference by external environmental factors (Goldstein et al., 2020; Gyllenhammer et al., 2022). Studies have found that maternal infection with bacteria, viruses, or other pathogenic microorganisms during pregnancy exposes the embryo to an inflammatory environment, leading to behavioral abnormalities in offspring from an early age (Wang X. et al., 2020; Ni et al., 2022; Zhang et al., 2022). LPS, a component of the cell wall of Gram-negative bacteria, can elicit inflammatory responses and is commonly used to generate models of maternal immune activation (Fernandez de Cossio et al., 2021). One study showed that offspring Sprague–Dawley rats exposed to LPS *in utero* on GDs 8, 10, and 12 displayed spatial learning and memory deficits in the MWM test (Hao et al., 2010). Meanwhile, a different study demonstrated that object recognition performance was poor in the offspring of LPS-challenged Long–Evans dams (Paris et al., 2011). In the current study, we found that offspring C57BL/6 J mice exposed to LPS prenatally took significantly longer and swam significantly longer distances to locate the hidden platform during the learning phase of the MWM test; in the memory phase of the trial, meanwhile, mice in the MLPS group displayed a decrease in the distance swam and time spent percent in the target quadrant, indicating that maternal immune activation resulted in cognitive deficits. This result is consistent with our previous study, in which we showed that aged offspring CD-1 mice of LPS-treated mothers exhibited cognitive impairment, as determined in the MWM test (Zhang et al., 2020).

Resveratrol has been reported to exert a protective effect against cognitive dysfunction in several animal models of neuropsychiatric disorders. For instance, resveratrol was shown to ameliorate short-term cognitive impairment in aged mice following surgery under local anesthesia (Wang et al., 2018). Additionally, the cognitive deficits induced by the anesthetic isoflurane in aged mice were abolished by pretreatment with resveratrol (Li et al., 2014). In full agreement with our previous studies, our current results indicated that resveratrol effectively improved maternal LPS exposure-induced learning and memory impairment in offspring mice as evidenced by the shortened latency and reduced distance in the trial phase of the MWM test and the increase in the time and distance percent in the target quadrant in the retention phase of the test among mice in the MLPS + Res group relative to that seen in animals of the MLPS group.

### 4.2 Resveratrol improved the response to inflammation induced by maternal immune activation

Elevated pro-inflammatory cytokine levels in the hippocampus were closely associated with cognitive impairment. Maternal exposure to LPS during the late stages of gestation led to an increase in inflammatory cytokine concentrations through a series of downstream signaling pathways. These cytokines could cross the maternal–placenta barrier and induce an inflammatory response in the fetus (Hisada et al., 2023). Previous studies have demonstrated that maternal exposure to LPS induced the activation of microglia/macrophages and stimulated the release of inflammatory cytokines such as IL-1β, IL-6, and TNF-α in the brains of offspring (Dominguez Rubio et al., 2017; Rahimi et al., 2020). Our recent work suggested that maternal exposure to LPS significantly upregulated inflammatory cytokine levels in the blood, which was accompanied by cognitive decline in the offspring (Ni et al., 2022; Zhang et al., 2022). In the current study, compared with the Control group, the hippocampal levels of IL-1β, IL-6, and TNF-α were increased in the MLPS group, which likely contributed to the cognitive impairment induced by maternal LPS exposure. Moreover, it has been reported that environmental enrichment could ameliorate the inflammatory response induced by maternal immune activation, as well as reverse the consequent cognitive impairment (Zhao et al., 2020). Resveratrol was effective in mitigating the cognitive decline in aging rats by inhibiting the production of pro-inflammatory cytokines (Gocmez et al., 2016). Here, we found that resveratrol reversed the increase in pro-inflammatory cytokine concentrations induced by prenatal exposure to LPS, suggesting that resveratrol treatment improved the inflammatory response caused by maternal immune activation.

### 4.3 Resveratrol improved the synaptic dysfunction induced by maternal immune activation

Hippocampal synaptic plasticity is a key biological mechanism underpinning learning and memory at the cellular level (Fuchsberger and Paulsen, 2022). Synaptic plasticity-associated proteins, including BDNF, PSD-95, and SYP, play an essential role in the regulation of dendrite growth and synaptic vesicle release (Chen et al., 2018; Liu et al., 2023). Animal experiments have suggested that maternal LPS exposure can disrupt synaptic pruning, neural circuit formation, and synaptic plasticity in the hippocampus (Xiao et al., 2021). Baharnoori et al. (2009) reported that dendritic spine numbers and dendritic length were decreased in the brains of offspring of mothers exposed to LPS during pregnancy. Meanwhile, Hao et al. (2010) revealed that maternal LPS exposure downregulated the expression of SYP and increased that of glial fibrillary acidic protein (GFAP) in the hippocampal CA1 region of offspring mice (Hao et al., 2010). SIRT1, a member of the class III family of histone acetylases, is known to positively regulate the expression of BDNF by restricting miR-134 levels via a repressor complex containing the transcription factor YY1, and its activity is also related to cognitive function (Gao et al., 2010). The downregulation of SIRT1 and BDNF expression was reported to impair cognitive function in a rat model of schizophrenia (Niu et al., 2020). The results of the current study showed that maternal LPS treatment significantly decreased the expression levels of SIRT1, BDNF, PSD-95, and SYP in the offspring, suggesting that impaired synaptic plasticity may underlie the cognitive deficits induced by maternal immune activation, likely due to SIRT1/BDNF signaling dysregulation. Furthermore, consistent with its function as an effective SIRT1 activator, resveratrol reversed the decrease in SIRT1, BDNF, PSD-95, and SYP expression levels and alleviated the learning and memory impairment caused by maternal LPS treatment. These results are in accordance with those of previous research that showed that resveratrol treatment reversed chronic unpredictable mild stress-induced cognitive impairment in rats by modulating the SIRT1/BDNF pathway in the hippocampus (Shen et al., 2018).

### 4.4 Correlations between the parameters of the MWM test and the markers of inflammation response and synaptic plasticity

A large body of evidence suggests that the levels of pro-inflammatory cytokines and synaptic plasticity-associated proteins in the hippocampus are closely related to learning and memory function. Elevated pro-inflammatory cytokine counts in peripheral blood have been correlated with cognitive dysfunction in aging mice that were subjected to LPS exposure *in utero* (Ni et al., 2022). Here, we found that hippocampal pro-inflammatory cytokine levels were positively correlated with the latency and distance in the training phase and negatively correlated with the time spent and distance percent in the probe trial in the MWM test, suggestive of a link between central inflammation and maternal LPS exposure-induced learning and memory impairment. In our previous study, using a mouse model of maternal sleep deprivation, we revealed that reduced levels of BDNF, PSD-95, and SYP in the hippocampus were strongly correlated with cognitive impairment (Zhang et al., 2023). Here, our results suggested that the maternal LPS exposure-induced decrease in the levels of SIRT1, BDNF, PSD-95, and SYP were correlated with the parameters of the MWM test.

Our study had several limitations. Firstly, we only examined the effects of maternal LPS exposure on male offspring and did not investigate whether maternal exposure to inflammation exerts sex-dependent behavioral effects. Secondly, we did not assess whether SIRT1 mediates the protective effect of resveratrol on cognition. Thirdly, we did not directly evaluate the effects of resveratrol treatment on dendrite morphology (dendritic density and dendrite length) or synaptic plasticity following exposure to LPS. Fourthly, we did not use immunohistochemical techniques to localize synaptic proteins (PSD-95, SYP, and BDNF) in different regions of the hippocampus. Finally, we performed only the MWM test and no other hippocampus-dependent cognitive tests.

## 5 Conclusion

Collectively, our findings demonstrated that resveratrol administration considerably improved learning and memory impairment in offspring mice of mothers exposed to LPS during gestation. A decrease in pro-inflammation cytokine concentrations and an increase in the expression levels of SIRT1, BDNF, PSD-95, and SYP in the hippocampus following resveratrol treatment contributed to reducing the cognitive deficits in offspring mice resulting from maternal immune activation. Our results suggest that resveratrol has potential as a treatment for cognitive impairment.

## Data availability statement

The original contributions presented in the study are included in the article/supplementary material, further inquiries can be directed to the corresponding authors.

## Ethics statement

The animal study was approved by all animal experiments complied with the guidelines for humane treatment established by the Association of Laboratory Animal Sciences and the Center for Laboratory Animal Sciences of the Anhui Medical University (No. LLSC20190710). The study was conducted in accordance with the local legislation and institutional requirements.

## Author contributions

Y-MZ: Conceptualization, Data curation, Formal analysis, Investigation, Methodology, Writing – original draft, Writing – review & editing. R-MW: Conceptualization, Data curation, Investigation, Methodology, Writing – original draft, Writing – review & editing. M-YZ: Data curation, Formal analysis, Investigation, Methodology, Software, Writing – original draft, Writing – review & editing. K-XZ: Conceptualization, Data curation, Investigation, Methodology, Software, Validation, Writing – original draft. J-YZ: Conceptualization, Data curation, Investigation, Methodology, Software, Writing – original draft. S-KF: Conceptualization, Data curation, Formal analysis, Investigation, Methodology, Writing – original draft. Y-JG: Conceptualization, Data curation, Investigation, Methodology, Writing – review & editing. X-YK: Conceptualization, Data curation, Formal analysis, Investigation, Project administration, Writing – review & editing. G-HC: Conceptualization, Data curation, Funding acquisition, Investigation, Writing – review & editing. X-YL: Data curation, Formal analysis, Funding acquisition, Resources, Software, Validation, Visualization, Project administration, Supervision, Writing – review & editing.

## References

[ref1] Baharnoori M. Brake W. G. Srivastava L. K. (2009). Prenatal immune challenge induces developmental changes in the morphology of pyramidal neurons of the prefrontal cortex and hippocampus in rats. Schizophr. Res. 107, 99–109. doi: 10.1016/j.schres.2008.10.003, PMID: 19004618

[ref2] Bock J. Wainstock T. Braun K. Segal M. (2015). Stress in utero: prenatal programming of brain plasticity and cognition. Biol. Psychiatry 78, 315–326. doi: 10.1016/j.biopsych.2015.02.036, PMID: 25863359

[ref3] Buglio D. S. Marton L. T. Laurindo L. F. Guiguer E. L. Araujo A. C. Buchaim R. L.. (2022). The role of resveratrol in mild cognitive impairment and Alzheimer’s disease: a systematic review. J. Med. Food 25, 797–806. doi: 10.1089/jmf.2021.0084, PMID: 35353606

[ref4] Chen J. Niu Q. Xia T. Zhou G. Li P. Zhao Q.. (2018). ERK1/2-mediated disruption of BDNF-Trk B signaling causes synaptic impairment contributing to fluoride-induced developmental neurotoxicity. Toxicology 410, 222–230. doi: 10.1016/j.tox.2018.08.009, PMID: 30130557

[ref5] Dominguez Rubio A. P. Correa F. Aisemberg J. Dorfman D. Bariani M. V. Rosenstein R. E.. (2017). Maternal administration of melatonin exerts short- and long-term neuroprotective effects on the offspring from lipopolysaccharide-treated mice. J. Pineal Res. 63:e12439. doi: 10.1111/jpi.12439, PMID: 28776755

[ref6] Elibol-Can B. Kilic E. Yuruker S. Jakubowska-Dogru E. (2014). Investigation into the effects of prenatal alcohol exposure on postnatal spine development and expression of synaptophysin and PSD95 in rat hippocampus. Int. J. Dev. Neurosci. 33, 106–114. doi: 10.1016/j.ijdevneu.2013.12.003, PMID: 24365761

[ref7] Fernandez de Cossio L. Lacabanne C. Bordeleau M. Castino G. Kyriakakis P. Tremblay M. E. (2021). Lipopolysaccharide-induced maternal immune activation modulates microglial CX3CR1 protein expression and morphological phenotype in the hippocampus and dentate gyrus, resulting in cognitive inflexibility during late adolescence. Brain Behav. Immun. 97, 440–454. doi: 10.1016/j.bbi.2021.07.025, PMID: 34343619

[ref8] Fuchsberger T. Paulsen O. (2022). Modulation of hippocampal plasticity in learning and memory. Curr. Opin. Neurobiol. 75:102558. doi: 10.1016/j.conb.2022.102558 35660989

[ref9] Gaitan J. M. Moon H. Y. Stremlau M. Dubal D. B. Cook D. B. Okonkwo O. C.. (2021). Effects of aerobic exercise training on systemic biomarkers and cognition in late middle-aged adults at risk for Alzheimer’s disease. Front. Endocrinol. 12:660181. doi: 10.3389/fendo.2021.660181, PMID: 34093436 PMC8173166

[ref10] Gao J. Wang W. Y. Mao Y. W. Graff J. Guan J. S. Pan L.. (2010). A novel pathway regulates memory and plasticity via SIRT1 and miR-134. Nature 466, 1105–1109. doi: 10.1038/nature09271, PMID: 20622856 PMC2928875

[ref11] Gąssowska M. Baranowska-Bosiacka I. Moczydłowska J. Frontczak-Baniewicz M. Gewartowska M. Strużynska L.. (2016). Perinatal exposure to lead (Pb) induces ultrastructural and molecular alterations in synapses of rat offspring. Toxicology 373, 13–29. doi: 10.1016/j.tox.2016.10.014, PMID: 27974193

[ref12] Gocmez S. S. Gacar N. Utkan T. Gacar G. Scarpace P. J. Tumer N. (2016). Protective effects of resveratrol on aging-induced cognitive impairment in rats. Neurobiol. Learn. Mem. 131, 131–136. doi: 10.1016/j.nlm.2016.03.022, PMID: 27040098

[ref13] Goldstein J. A. Gallagher K. Beck C. Kumar R. Gernand A. D. (2020). Maternal-fetal inflammation in the placenta and the developmental origins of health and disease. Front. Immunol. 11:531543. doi: 10.3389/fimmu.2020.531543, PMID: 33281808 PMC7691234

[ref14] Gomes B. A. Q. Silva J. P. B. Romeiro C. F. R. Dos Santos S. M. Rodrigues C. A. Gonçalves P. R.. (2018). Neuroprotective mechanisms of resveratrol in Alzheimer’s disease: role of SIRT1. Oxidative Med. Cell. Longev. 2018:8152373. doi: 10.1155/2018/8152373, PMID: 30510627 PMC6232815

[ref15] Gyllenhammer L. E. Rasmussen J. M. Bertele N. Halbing A. Entringer S. Wadhwa P. D.. (2022). Maternal inflammation during pregnancy and offspring brain development: the role of mitochondria. Biol. Psychiatry Cogn. Neurosci. Neuroimaging 7, 498–509. doi: 10.1016/j.bpsc.2021.11.003, PMID: 34800727 PMC9086015

[ref16] Han V. X. Patel S. Jones H. F. Dale R. C. (2021). Maternal immune activation and neuroinflammation in human neurodevelopmental disorders. Nat. Rev. Neurol. 17, 564–579. doi: 10.1038/s41582-021-00530-8, PMID: 34341569

[ref17] Hao L. Y. Hao X. Q. Li S. H. Li X. H. (2010). Prenatal exposure to lipopolysaccharide results in cognitive deficits in age-increasing offspring rats. Neuroscience 166, 763–770. doi: 10.1016/j.neuroscience.2010.01.006, PMID: 20074621

[ref18] Hisada C. Kajimoto K. Tsugane H. Mitsuo I. Azuma K. Kubo K. Y. (2023). Maternal chewing alleviates prenatal stress-related neuroinflammation mediated by microglia in the hippocampus of the mouse offspring. J. Prosthodont. Res. 67, 588–594. doi: 10.2186/jpr.JPR_D_22_00255, PMID: 36792221

[ref19] Kentner A. C. Bilbo S. D. Brown A. S. Hsiao E. Y. McAllister A. K. Meyer U.. (2019). Maternal immune activation: reporting guidelines to improve the rigor, reproducibility, and transparency of the model. Neuropsychopharmacology 44, 245–258. doi: 10.1038/s41386-018-0185-7, PMID: 30188509 PMC6300528

[ref20] Knuesel I. Chicha L. Britschgi M. Schobel S. A. Bodmer M. Hellings J. A.. (2014). Maternal immune activation and abnormal brain development across CNS disorders. Nat. Rev. Neurol. 10, 643–660. doi: 10.1038/nrneurol.2014.187, PMID: 25311587

[ref21] Kuypers E. Jellema R. K. Ophelders D. R. Dudink J. Nikiforou M. Wolfs T. G.. (2013). Effects of intra-amniotic lipopolysaccharide and maternal betamethasone on brain inflammation in fetal sheep. PLoS One 8:e81644. doi: 10.1371/journal.pone.0081644, PMID: 24358119 PMC3866104

[ref22] Kwon H. K. Choi G. B. Huh J. R. (2022). Maternal inflammation and its ramifications on fetal neurodevelopment. Trends Immunol. 43, 230–244. doi: 10.1016/j.it.2022.01.007, PMID: 35131181 PMC9005201

[ref23] Li X. M. Zhou M. T. Wang X. M. Ji M. H. Zhou Z. Q. Yang J. J. (2014). Resveratrol pretreatment attenuates the isoflurane-induced cognitive impairment through its anti-inflammation and -apoptosis actions in aged mice. J. Mol. Neurosci. 52, 286–293. doi: 10.1007/s12031-013-0141-2, PMID: 24126892

[ref24] Liu B. Kou J. Li F. Huo D. Xu J. Zhou X.. (2020). Lemon essential oil ameliorates age-associated cognitive dysfunction via modulating hippocampal synaptic density and inhibiting acetylcholinesterase. Aging 12, 8622–8639. doi: 10.18632/aging.103179, PMID: 32392535 PMC7244039

[ref25] Liu W. Y. Li Y. Li Y. Xu L. Z. Jia J. P. (2023). Carnosic acid attenuates AβOs-induced apoptosis and synaptic impairment via regulating NMDAR2B and its downstream cascades in SH-SY5Y cells. Mol. Neurobiol. 60, 133–144. doi: 10.1007/s12035-022-03032-w, PMID: 36224322

[ref26] Liu Y. Zhang Y. Zheng X. Fang T. Yang X. Luo X.. (2018). Galantamine improves cognition, hippocampal inflammation, and synaptic plasticity impairments induced by lipopolysaccharide in mice. J. Neuroinflammation 15:112. doi: 10.1186/s12974-018-1141-5, PMID: 29669582 PMC5907415

[ref27] Moussa C. Hebron M. Huang X. Ahn J. Rissman R. A. Aisen P. S.. (2017). Resveratrol regulates neuro-inflammation and induces adaptive immunity in Alzheimer’s disease. J. Neuroinflammation 14:1. doi: 10.1186/s12974-016-0779-0, PMID: 28086917 PMC5234138

[ref28] Murack M. Smith K. B. Traynor O. H. Pirwani A. F. Gostlin S. K. Mohamed T.. (2023). Environmental enrichment alters LPS-induced changes in BDNF and PSD-95 expressions during puberty. Brain Res. 1806:148283. doi: 10.1016/j.brainres.2023.148283 36801452

[ref29] Ni M. Z. Zhang Y. M. Li Y. Wu Q. T. Zhang Z. Z. Chen J.. (2022). Environmental enrichment improves declined cognition induced by prenatal inflammatory exposure in aged CD-1 mice: role of NGPF2 and PSD-95. Front. Aging Neurosci. 14:1021237. doi: 10.3389/fnagi.2022.1021237, PMID: 36479357 PMC9720164

[ref30] Niu J. Cao Y. Ji Y. (2020). Resveratrol, a SIRT1 activator, ameliorates MK-801-induced cognitive and motor impairments in a neonatal rat model of schizophrenia. Front. Psych. 11:716. doi: 10.3389/fpsyt.2020.00716 PMC7393240 32793005

[ref31] O’Donnell K. J. Meaney M. J. (2017). Fetal origins of mental health: the developmental origins of health and disease hypothesis. Am. J. Psychiatry 174, 319–328. doi: 10.1176/appi.ajp.2016.16020138 27838934

[ref32] Oldenburg K. S. O’Shea T. M. Fry R. C. (2020). Genetic and epigenetic factors and early life inflammation as predictors of neurodevelopmental outcomes. Semin. Fetal Neonatal Med. 25:101115. doi: 10.1016/j.siny.2020.101115 32444251 PMC7363586

[ref33] Osborne L. M. Yenokyan G. Fei K. Kraus T. Moran T. Monk C.. (2019). Innate immune activation and depressive and anxious symptoms across the peripartum: an exploratory study. Psychoneuroendocrinology 99, 80–86. doi: 10.1016/j.psyneuen.2018.08.038, PMID: 30195110 PMC6234836

[ref34] Palasz E. Wysocka A. Gasiorowska A. Chalimoniuk M. Niewiadomski W. Niewiadomska G. (2020). BDNF as a promising therapeutic agent in Parkinson’s disease. Int. J. Mol. Sci. 21:1170. doi: 10.3390/ijms21031170, PMID: 32050617 PMC7037114

[ref35] Paris J. J. Brunton P. J. Russell J. A. Frye C. A. (2011). Immune stress in late pregnant rats decreases length of gestation and fecundity, and alters later cognitive and affective behaviour of surviving pre-adolescent offspring. Stress 14, 652–664. doi: 10.3109/10253890.2011.628719, PMID: 21995525 PMC3376536

[ref36] Pyo I. S. Yun S. Yoon Y. E. Choi J. W. Lee S. J. (2020). Mechanisms of aging and the preventive effects of resveratrol on age-related diseases. Molecules 25:4649. doi: 10.3390/molecules25204649, PMID: 33053864 PMC7587336

[ref37] Rahimi S. Peeri M. Azarbayjani M. A. Anoosheh L. Ghasemzadeh E. Khalifeh N.. (2020). Long-term exercise from adolescence to adulthood reduces anxiety- and depression-like behaviors following maternal immune activation in offspring. Physiol. Behav. 226:113130. doi: 10.1016/j.physbeh.2020.113130, PMID: 32791182

[ref38] Segovia S. A. Vickers M. H. Reynolds C. M. (2017). The impact of maternal obesity on inflammatory processes and consequences for later offspring health outcomes. J. Dev. Orig. Health Dis. 8, 529–540. doi: 10.1017/S2040174417000204, PMID: 28343461

[ref39] Shen J. Xu L. Qu C. Sun H. Zhang J. (2018). Resveratrol prevents cognitive deficits induced by chronic unpredictable mild stress: Sirt 1/miR-134 signalling pathway regulates CREB/BDNF expression in hippocampus in vivo and in vitro. Behav. Brain Res. 349, 1–7. doi: 10.1016/j.bbr.2018.04.050, PMID: 29715537

[ref40] Wang X. Fang H. Xu G. Yang Y. Xu R. Liu Q.. (2020). Resveratrol prevents cognitive impairment in type 2 diabetic mice by upregulating Nrf 2 expression and transcriptional level. Diabetes Metab. Syndr. Obes. 13, 1061–1075. doi: 10.2147/DMSO.S243560, PMID: 32308456 PMC7150671

[ref41] Wang B. Ge S. Xiong W. Xue Z. (2018). Effects of resveratrol pretreatment on endoplasmic reticulum stress and cognitive function after surgery in aged mice. BMC Anesthesiol. 18:141. doi: 10.1186/s12871-018-0606-5, PMID: 30305045 PMC6180510

[ref42] Wang R. Wu Z. Liu M. Wu Y. Li Q. Ba Y.. (2021). Resveratrol reverses hippocampal synaptic markers injury and SIRT1 inhibition against developmental Pb exposure. Brain Res. 1767:147567. doi: 10.1016/j.brainres.2021.147567 34175265

[ref43] Wang F. Zhang Z. Z. Cao L. Yang Q. G. Lu Q. F. Chen G. H. (2020). Lipopolysaccharide exposure during late embryogenesis triggers and drives Alzheimer-like behavioral and neuropathological changes in CD-1 mice. Brain Behav. 10:e01546. doi: 10.1002/brb3.1546, PMID: 31997558 PMC7066339

[ref44] Wang R. Zhang Y. Li J. Zhang C. (2017). Resveratrol ameliorates spatial learning memory impairment induced by Aβ1-42 in rats. Neuroscience 344, 39–47. doi: 10.1016/j.neuroscience.2016.08.051, PMID: 27600946

[ref45] Xiao L. Yan J. Feng D. Ye S. Yang T. Wei H.. (2021). Critical role of TLR4 on the microglia activation induced by maternal LPS exposure leading to ASD-like behavior of offspring. Front. Cell Dev. Biol. 9:634837. doi: 10.3389/fcell.2021.634837, PMID: 33748121 PMC7969707

[ref46] Yang A. J. T. Bagit A. Mac Pherson R. E. K. (2021). Resveratrol, metabolic dysregulation, and Alzheimer’s disease: considerations for neurogenerative disease. Int. J. Mol. Sci. 22:4628. doi: 10.3390/ijms22094628, PMID: 33924876 PMC8125227

[ref47] Zhang Y. M. Wei R. M. Li X. Y. Feng Y. Z. Zhang K. X. Ge Y. J.. (2023). Long-term environmental enrichment overcomes depression, learning, and memory impairment in elderly CD-1 mice with maternal sleep deprivation exposure. Front. Aging Neurosci. 15:1177250. doi: 10.3389/fnagi.2023.1177250 37168717 PMC10164971

[ref48] Zhang Z. Z. Zeng L. P. Chen J. Wu Y. F. Wang Y. T. Xia L.. (2022). Long-term environmental enrichment relieves dysfunctional cognition and synaptic protein levels induced by prenatal inflammation in older CD-1 mice. Neural Plast. 2022:1483101. doi: 10.1155/2022/1483101, PMID: 35574247 PMC9106518

[ref49] Zhang L. Zhao Q. Chen C. H. Qin Q. Z. Zhou Z. Yu Z. P. (2014). Synaptophysin and the dopaminergic system in hippocampus are involved in the protective effect of rutin against trimethyltin-induced learning and memory impairment. Nutr. Neurosci. 17, 222–229. doi: 10.1179/1476830513Y.0000000085, PMID: 24001577

[ref50] Zhang Z. Z. Zhuang Z. Q. Sun S. Y. Ge H. H. Wu Y. F. Cao L.. (2020). Effects of prenatal exposure to inflammation coupled with stress exposure during adolescence on cognition and synaptic protein levels in aged CD-1 mice. Front. Aging Neurosci. 12:157. doi: 10.3389/fnagi.2020.00157, PMID: 32774299 PMC7381390

[ref51] Zhao X. Rondón-Ortiz A. N. Lima E. P. Puracchio M. Roderick R. C. Kentner A. C. (2020). Therapeutic efficacy of environmental enrichment on behavioral, endocrine, and synaptic alterations in an animal model of maternal immune activation. Brain Behav. Immun. Health 3:100043. doi: 10.1016/j.bbih.2020.100043, PMID: 32368757 PMC7197879

[ref52] Zhuang Z. Q. Zhang Z. Z. Zhang Y. M. Ge H. H. Sun S. Y. Zhang P.. (2020). A long-term enriched environment ameliorates the accelerated age-related memory impairment induced by gestational administration of lipopolysaccharide: role of plastic mitochondrial quality control. Front. Cell. Neurosci. 14:559182. doi: 10.3389/fncel.2020.559182, PMID: 33613195 PMC7886998

